# Natural products for the treatment of ulcerative colitis: focus on the JAK/STAT pathway

**DOI:** 10.3389/fimmu.2025.1538302

**Published:** 2025-02-26

**Authors:** Dan Long, Chenhan Mao, Wei Zhang, Ying Zhu, Yin Xu

**Affiliations:** ^1^ Department of Gastroenterology, The First Hospital of Hunan University of Chinese Medicine, Changsha, Hunan, China; ^2^ Affiliated Hospital of Integrated Traditional Chinese and Western Medicine, Nanjing University of Chinese Medicine, Nanjing, Jiangsu, China

**Keywords:** Janus kinase (JAK), signal transducer and activator of transcription (STAT), ulcerative colitis, natural products, traditional herbal medicine

## Abstract

Ulcerative colitis (UC) is an autoimmune disease with an incompletely understood pathogenesis. The Janus kinase (JAK)/signal transducer and activator of transcription (STAT) signaling pathway plays a key role in immune response and inflammation. More and more studies demonstrated that JAK/STAT signaling pathway is associated with the pathogenesis of UC. The JAK/STAT pathway affects UC in multiple ways by regulating intestinal inflammatory response, affecting intestinal mucosal barrier, modulating T cell homeostasis, and regulating macrophages. Encouragingly, natural products are promising candidates for the treatment of UC. Natural products have the advantage of being multi-targeted and rich in therapeutic modalities. This review summarized the research progress of JAK/STAT pathway-mediated UC. Furthermore, the latest studies on natural products targeting the JAK/STAT pathway for the treatment of UC were systematically summarized, including active ingredients such as arbutin, aloe polysaccharide, berberine, matrine, curcumin, Ginsenoside Rh2, and so on. The aim of this paper is to provide new ideas for drug development to regulate JAK/STAT signaling for treating UC.

## 1 Introduction

Ulcerative colitis (UC) is a refractory digestive disease defined by recurring and remitting mucosal inflammation. Common clinical signs of UC include recurring stomach pain, diarrhea, and hematochezia. Typical medications used clinically as the primary treatment option for UC include aminosalicylates, corticosteroids, immunosuppressants, biological agents, and microecologics (1). Despite the large number of drugs available for the treatment of UC, its treatment remains complex and challenging due to a variety of side effects, medication tolerance, and high relapse rates (2). Therefore, further development of more effective treatments for UC has become urgent.

The Janus tyrosine protein kinase (JAK)/signal transducer and activator of transcription (STAT) signaling pathway has been identified as a classical inflammatory pathway. The JAK/STAT signaling is involved in biological processes such as cell proliferation, differentiation, and apoptosis. The JAK/STAT pathway plays a key role in the immune response and has become a focus of research in autoimmune and inflammatory diseases (3). Notably, the JAK/STAT pathway is associated with damage induced by exaggerated an innate immune system response stimulated by immune checkpoint inhibitors (4). JAK/STAT signaling is frequently dysregulated in UC patients, indicating the importance of JAK/STAT regulation in UC (5, 6). Furthermore, in the colitis rat model, the severity of intestinal illness was positively associated to the expression of JAK2 and STAT3 (7). Theoretically, intervening in the JAK/STAT signaling pathway using safe and effective drugs may be an effective way to alleviate or treat UC. Currently, several JAK inhibitors have achieved efficacy in numerous clinical settings. The non-selective JAK inhibitor tofacitinib has been approved for moderate and severe UC (8). Encouragingly, natural products shows potential for the treatment of UC (9, 10). However, the existing studies are scattered and unsystematic. To our knowledge, this is the first thorough review that elaborates on recent advances of active ingredients in treating UC by modulating the JAK/STAT signaling pathway.

In this review, the current knowledge of the composition, activation, and regulation of the JAK/STAT pathway was discussed. Secondly, the role and mechanism of the JAK/STAT pathway in UC were particularly emphasized. Finally, we also systematically summarized the application of natural products targeting JAK/STAT signaling against UC. This review aims to provide new research ideas for traditional Chinese medicine (TCM) in the prevention and treatment of UC.

## 2 JAK/STAT pathway

### 2.1 Composition and activation of the JAK/STAT pathway

JAK is a non-receptor tyrosine protein kinase that is activated by numerous cytokines and initiates downstream target genes via STAT, which in turn regulates a variety of cellular functions (3).The JAK/STAT pathway consists of three main components, including tyrosine kinase-associated receptors, JAKs, and STATs. Four types of JAKs have been identified, including JAK1, JAK2, JAK3, and tyrosine kinase 2 (TYK2). Among them, JAK3 is expressed only in bone marrow and lymphocytes, while other members are widely found in various tissues and organs in the body (11). The JAK proteins are made up of FERM (the complex of four point one, ezrin, radixin, and moesin), Src homology domain (SH2), pseudokinase, and kinase domains. STAT proteins are downstream signaling molecules of JAK. STATs consist of seven members, namely STAT1, STAT2, STAT3, STAT4, STAT5A, STAT5B, and STAT6, which are widely distributed in various tissues. STATs proteins mainly contain five structural domains, including N-terminal conserved sequences, DNA-binding region, Src homology domain 3 (SH3) structural region, SH2 structural region, and C-terminal transcriptional activation sequence. The SH2 structural area of STATs is identical to the analogous core sequence in JAKs, which is in charge of recognizing individual JAKs. Cytokines attach to cell-surface receptors, which dimerize and stimulate the polymerization and phosphorylation of JAKs. Activated JAKs can then bind to the SH2 structural domain of STATs, which are activated by phosphorylation modification and ultimately enter the nucleus as homodimers or heterodimers, thus promoting transcription of specific target genes (12). STAT is then dephosphorylated in the nucleus and returned to the cytoplasm (12). Among the STAT family, STAT3 has been recognized to play a central role in signaling from the plasma membrane to the nucleus (13). STAT3 is activated by phosphorylation of tyrosine (Y705) or serine (S727) residues in the transactivation domains, creating a STAT3 dimer that moves into the nucleus, where it promotes the transcription of target genes. Phosphorylation of STAT3 at the Y705 site occurs predominantly through members of the JAK family, whereas phosphorylation at the S727 site is usually carried out by mitogen-activated protein kinase, cell cycle protein-dependent kinase 5, and protein kinase C.

### 2.2 Negative regulation of the JAK/STAT pathway

**Figure 1:**
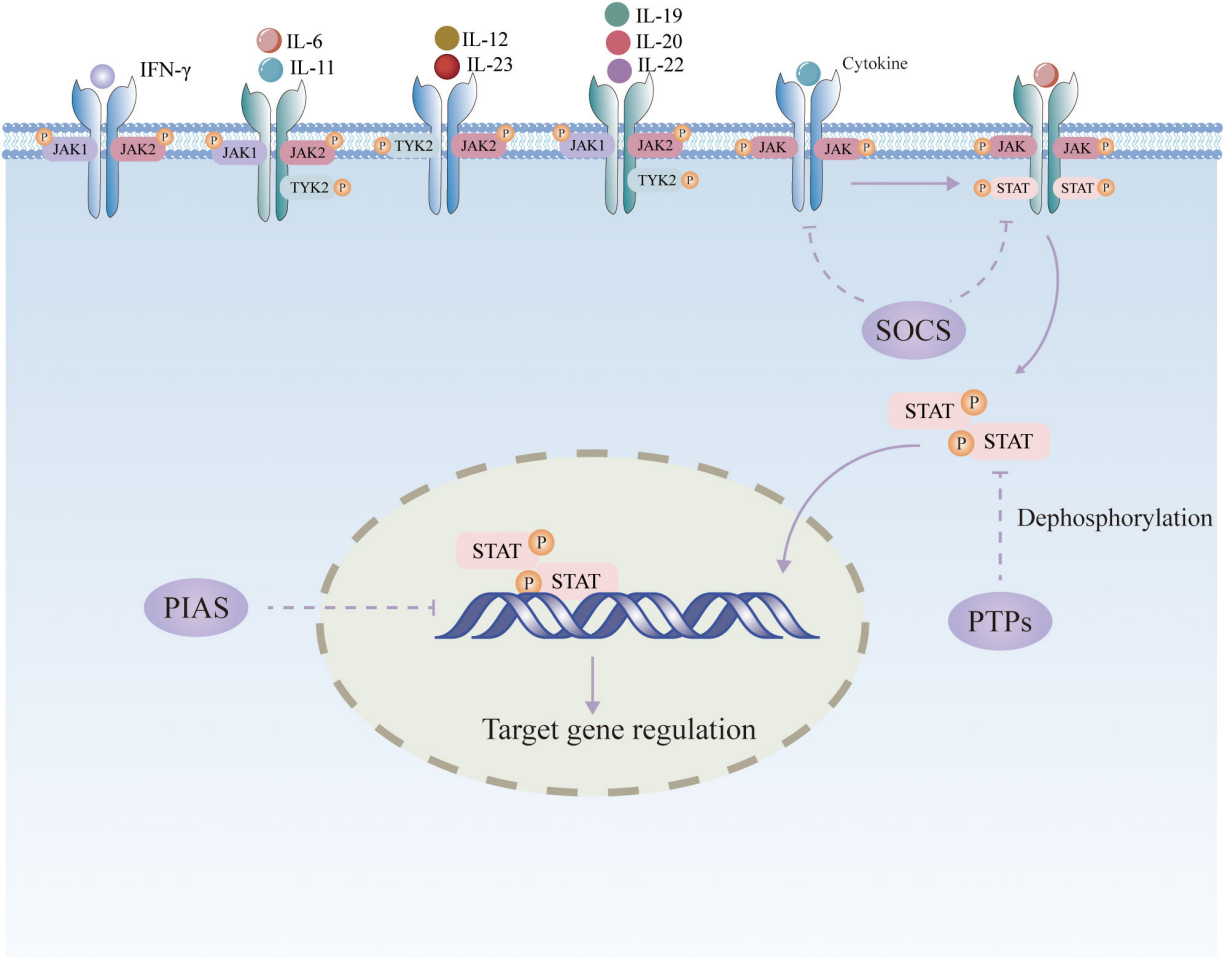
Schematic diagram of the JAK/STAT signaling pathway.

The JAK/STAT pathway is primarily regulated negatively by three types of factors: suppressor of cytokine signaling (SOCS), protein inhibitor of activated STAT (PIAS), and protein tyrosine phosphatase (PTP) (14). (**Figure 1**). The SOCS family is the main signaling molecule that weakens the JAK/STAT pathway, including CIS, SOCS1, SOCS2, SOCS3, SOCS4, SOCS5, SOCS6, and SOCS7. Activated STAT entering the nucleus promotes the transcription of SOCS, which has a negative regulatory effect on JAK/STAT signaling by inhibiting STAT receptor binding, inactivating JAK through N-terminal kinase inhibition, or binding and ubiquitinating JAK or STAT for proteasomal destruction (15). PIAS can interact with STAT to prevent STAT dimerization or prevent STAT dimers from binding to DNA. PTP can dephosphorylate JAK by interacting with receptors as a phosphatase. It can also directly dephosphorylate STAT dimers to block JAK/STAT signaling transmission (16).

### 2.3 JAK/STAT pathway and melanocortin system

The melanocortin system is a complex signaling system composed of multiple hormones, neuropeptides, and receptors, which exerts a widespread regulatory role in the physiological and pathological processes in the body. α-, β- and γ-melanocyte-stimulating hormone (MSH) and adrenocorticotropic hormone are important components of the melanocortin system. Melanocortin receptors (MCR, MC1R-MC5R) are important members of the G protein-coupled receptor superfamily. The latest clinical research data indicate that the expression of MC3R and MC5R is significantly increased in inflamed mucosa of inflammatory bowel disease (IBD) patients compared to normal mucosa (17). Importantly, the melanocortin system plays a key role in inflammation and immune regulation (18, 19). The melanocortin system is involved in the development of IBD through multiple pathways (20). Melanocortin peptides, especially α-MSH, have potent anti-inflammatory and immunomodulatory activities (21). It has been suggested that α-MSH may indirectly affect the activity of the JAK/STAT signaling pathway by regulating cytokine production (22, 23). Melanocortin attenuates myocardial ischemia/reperfusion injury by activating JAK/STAT signaling (24). Seemingly paradoxically, α-MSH was shown to activate the JAK2/STAT1 pathway by binding its MC5R receptor (25). The regulatory mechanism of melanocortin system on the JAK/STAT pathway remains to be further investigated in depth.

### 2.4 Cross-talk between the JAK/STAT pathway and other signaling networks

Diverse components of the JAK/STAT pathway, such as JAK, STAT, receptors, and gene transcription factors, are embedded in a dynamic cross-talk with other signaling networks. For example, the cross-talk between nuclear factor-kappa-B (NF-κB) and STAT3 has been observed in numerous inflammatory disorders and cancers. First, IL-6, a gene production regulated by NF-κB pathway, serves as a critical STAT3 activator (26). Second, STAT3-mediated acetylation of NF-κB p65 enhances its transcriptional activity in the nucleus and promotes the expression of pro-inflammatory factors such as IL-6 and TNF-α (27). Finally, STAT3 stimulates the expression of p52 and CD30, which induces sustained activation of non-canonical NF-κB signaling (28). Furthermore, dimerization of IL-6-type cytokine receptors not only activates the JAK/STAT signaling pathway, but also induces the mitogen-activated protein kinase (MAPK) cascade by recruiting SH2-domain-containing tyrosine phosphatase (SHP2) to tyrosine-phosphorylated gp130 and phosphorylating it in a JAK1-dependent manner. The phosphorylated SHP2 combines with the growth factor receptor-bound protein/Son of Sevenless (Grb2-SOS) complex, resulting in the activation of the Ras-Raf-MAPK pathway (29). Autophagy in epithelial cells is usually considered to exert a protective effect in UC (30). On the one hand, it was found that the activation of JAK2/STAT3 pathway directly suppressed the transcription of autophagy regulator Beclin-1, contributing to the inhibition of autophagy and the initiation of intestinal cell death (31). On the other hand, autophagy promotes IFN-γ-induced Jak2/STAT1 activation by inhibiting the expression of reactive oxygen species and SHP2 (32).

Other inflammatory pathways such as the NF-κB pathway also play important roles in inflammation, but the NF-κB pathway is widely involved in a variety of physiopathologic processes with relatively limited specificity. In contrast, the JAK/STAT pathway is more direct and critical in cytokine-mediated inflammatory signaling. Thus, targeting the JAK/STAT pathway enables more precise intervention in the inflammatory process of UC. Besides, the JAK/STAT pathway involves diverse cytokines and immune cells. Compared to this, the regulatory scope of NF-κB is relatively limited. Natural products may target the JAK/STAT pathway to comprehensively regulate the inflammatory response through multi-target effects. Importantly, JAK inhibitors (e.g., tofacitinib) have shown significant efficacy in the treatment of UC, further validating the feasibility of the JAK/STAT pathway as a therapeutic target (33). In addition, some inflammatory pathways have complex regulatory mechanisms, making intervention difficult. For example, there are multiple upstream and downstream kinases in the MAPK pathway, with complex interactions between members. Comprehensive intervention may induce more adverse effects. The JAK/STAT pathway is comparatively clear and its intervention by natural products has been more intensively studied. Consequently, JAK/STAT a privileged target for natural products in UC.

## 3 Role of JAK/STAT pathway in the pathogenesis of UC

### 3.1 Influence on inflammatory response

**Figure 2:**
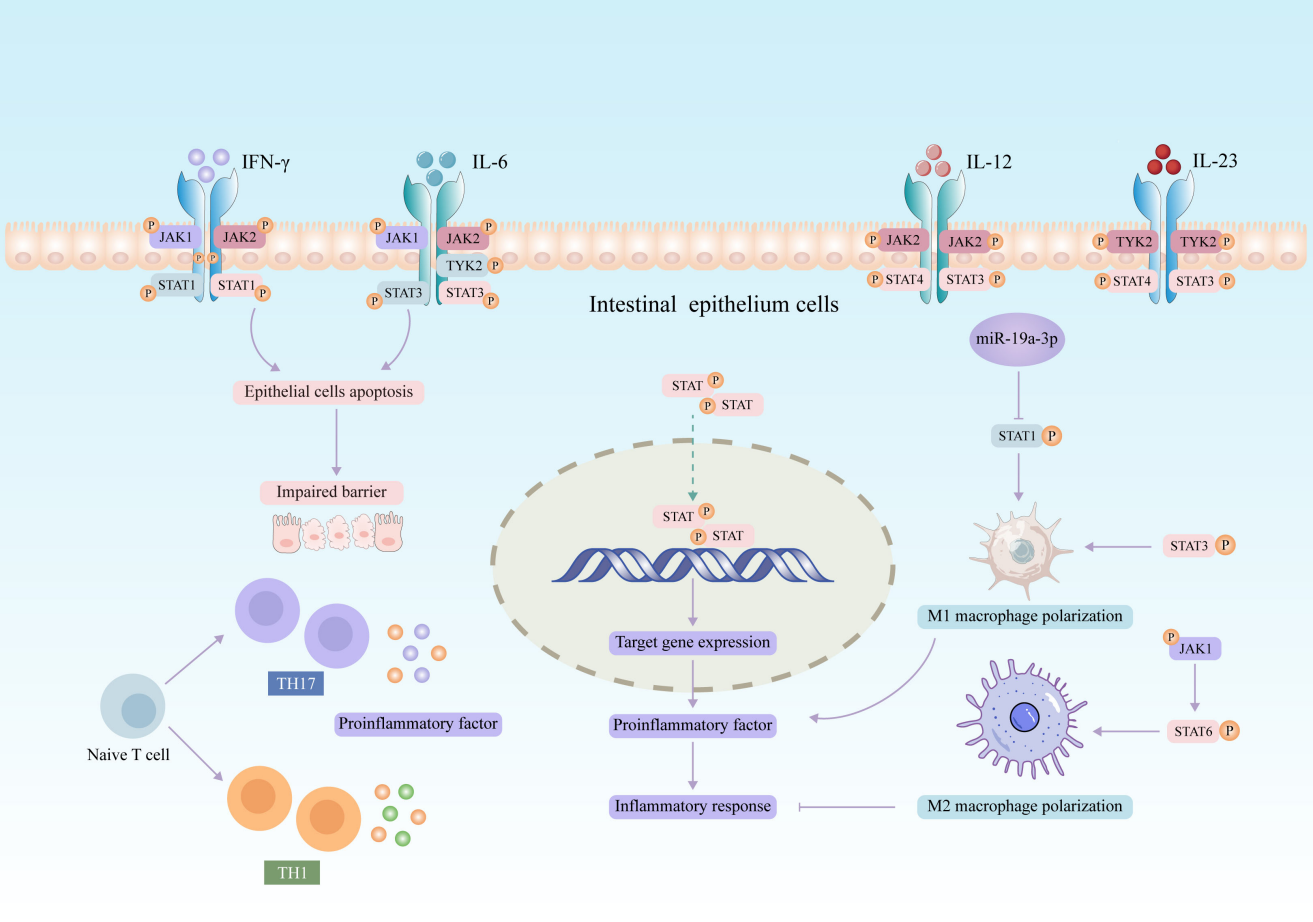
Potential role of JAK/STAT signaling pathway in the pathogenesis of UC.

The JAK2/STAT3 axis is a major pathway for transcription factors associated with mediating proinflammatory cytokine in intestinal mucosal inflammation. Inflammatory factors such as interferon-γ (IFN-γ) and interleukin (IL) have been found to promote the activation of JAK/STAT pathway, which in turn exerts immunomodulatory functions (34, 35). In recent years, increasing evidence suggested that aberrant activation of the JAK/STAT signaling pathway is related to the pathogenesis of UC. The expression of four JAK genes was upregulated in the intestinal mucosal epithelium of patients with active UC (36). Polymorphisms in JAK2 and STAT3 genes correlate with the severity of UC patients (37). Most cytokines mediate inflammatory responses by activating JAK/STAT pathway in UC (summarized in **Figure 2**). Previous studies have revealed that IL-6 is involved in the pathogenesis of UC (38). IL-6 binding to its receptor activates JAK1/2 and TYK2 and contributes to the phosphorylation and transcriptional activation of STAT3, which ultimately regulates T cell differentiation and inflammatory response (35, 39). Moreover, IL-12 and IL-23 activate STAT3 and STAT4 through JAK2 and TYK2, respectively (40, 41).

### 3.2 Impact on intestinal epithelial cells

Intestinal mucosal structures are maintained by a balance between apoptosis and proliferation of intestinal epithelial cells (IECs). However, IECs in UC patients exhibit a higher rate of apoptosis (42). Abnormal apoptosis leads to the loss of intestinal epithelial structures, disrupting the intestinal mucosal barrier and further activating excessive immune responses, eventually leading to uncontrollable inflammatory responses and mucosal damage. The JAK pathway is known to play an influential role in the regulation of cell proliferation and apoptosis (43). Studies showed that the activation of STAT1 or STAT3 promotes apoptosis in IECs (44, 45). miR-124-3p can directly target the STAT3 3’-UTR to modulate STAT3 expression (46). A recent study indicated that the overexpression of miR-124-3p attenuates apoptosis and reactive oxygen species production by targeting STAT3 in lipopolysaccharide (LPS)-induced colonocytes (47).

### 3.3 Modulation of intestinal macrophages

Under homeostatic conditions, circulating lymphocyte antigen 6 complex (Ly6C) monocytes in mice and CD14 monocytes in humans are constantly attracted to the intestinal tract and differentiate into mature F4/80 macrophages with high levels of CX3C chemokine receptor 1 (CX3CR1^hi^). CX3CR1 macrophages directly activate neighboring T cells to amplify the inflammatory response (48). A recent study found that gut-resident CX3CR1^hi^ macrophages trigger tertiary lymphoid structures and IgA response in situ (49). Furthermore, intestinal mucosal CXCR4^+^ IgG plasma cells drive the activation of CD4 macrophage and exacerbate UC (50). Compared to the lamina propria of the normal mucosa, the number of macrophages is greatly increased and activated in the localized colonic tissues of individuals with active UC, indicating that intestinal macrophages are implicated in the occurrence and progression of UC (51). Particularly, different expression of Tim-4 and CD4 can divide intestinal macrophages into three subsets, including locally maintained macrophages (Tim-4^+^CD4^+^), circulating monocyte-renewing macrophages (Tim-4^-^CD4^+^), and macrophages with the high monocyte-replenishment rate (Tim-4^-^CD4^-^) (52). Furthermore, macrophages from colonic lamina propria cells can be divided into subpopulations based on the expression of F4/80 and CD11b. F4/80^hi^ macrophages are considered to be intestinal resident macrophages, whereas CD11b^hi^ macrophages are regarded as infiltrative macrophages supplemented by circulating monocytes (53, 54). Interestingly, JAK/STAT signaling has an important regulatory effect on macrophage (55–58). It was found that inhibition of the JAK2/STAT3 pathway resulted in a significant reduction in apoptosis, collagen deposition, and immunoreactivity of intestinal macrophages (59). Notably, the levels of IFN-γ are markedly elevated in the mucosa of IBD patients. This cytokine promotes the pro-inflammatory characteristics of CD14^hi^ macrophages in humans (60). Consistently, the complete deletion of IFNγR1 or its downstream transcription factor STAT1 suppresses the formation of immature Ly6C MHCII macrophages (61).

Macrophages are highly plastic in different environments, exhibiting different phenotypes and functions depending on microenvironmental stimuli and signals (62). Macrophages are divided into classically activated M1-type macrophages with proinflammatory effects and alternatively activated M2-type macrophages with anti-inflammatory effects (63, 64), both of which are involved in UC pathology (65). Recently, a growing amount of studies indicated that STAT1 plays a critical role in the modulation of M1 macrophage polarization (66–68). It was reported that miR-19a-3p inhibited M1 macrophage polarization as an upstream regulator of STAT1 (69). Moreover, SOCS3-deficient macrophages showed increased STAT3 expression and M1 polarization (70). Interestingly, the JAK1/STAT6 pathway is an important pathway in the induction of M2 macrophage polarization (71). IL-4 binds to its receptor to activate JAK1, which recruits STAT6 phosphorylation and promotes the expression of M2 macrophage markers (72).

Notably, most of the current studies have been conducted mainly using *in vitro* bone marrow-derived macrophages (BMDMs). When cultured *in vitro*, BMDMs rely on specific cytokines to induce differentiation. However, the induction process is significantly different from the complex intestinal microenvironment *in vivo*. Moreover, BMDMs differ from intestinal macrophages in their degree of differentiation and maturation, leading to their distinct performance in the expression and activity of some key functional proteins. Apart from participating in immune defense, intestinal macrophages also play a crucial role in maintaining intestinal homeostasis and regulating intestinal microbiota balance. On the other hand, BMDMs lack gut-related signaling stimulation in the *in vitro* environment to fully exhibit these complex functions of intestinal macrophages.

### 3.4 Regulation of T cell balance

T cells are another important type of immune cells involved in adaptive immunity. Studies demonstrated that the JAK/STAT signaling pathway is critical in modulating T cell differentiation (35, 73, 74). A recent study showed a significant increase in the number of CD4 T cells in UC patients (75). Naive CD4 T cells are induced to differentiate into different types of T cells in different cytokine microenvironments, including T helper cell 1 (Th1), Th2, Th17, and regulatory T cells (Tregs). Abnormally activated CD4 T cells differentiate into subpopulations of Th1 and/or Th17 cells that subsequently infiltrate the colon to mediate autoimmune responses in UC (76). Cytokines such as Th1-induced IL-2 and IFN-γ, and Th17-induced IL-17 and IL-21 promote inflammatory responses and exacerbate colitis (77). In contrast, Tregs control effector T cell immunosuppression through intercellular contacts or secretion of anti-inflammatory cytokines. Th17/Treg balance facilitates the maintenance of intestinal immune homeostasis, an imbalance of which is the source of immune dysfunction in intestinal mucosa (78). Increasing data suggested that proinflammatory cytokines that stimulate the JAK/STAT signaling pathway govern the differentiation of naive Th1 and Th17 cell subsets and aggravate the development of UC (35, 73). STAT5 and forkhead box P3 (Foxp3) are key transcription factors for Tregs, whereas retinoic acid-related orphan receptor γt (RORγt) and STAT3 are key transcription factors for Th17 cells (79, 80). The overactivation of STAT3 promotes the Th17-like transformation of Treg and exacerbates immune responses (81). IL-12 or IFN-γ binds to their receptors to activate STAT1, STAT4, and the T-box transcription factor, driving the differentiation and function of Th1 cells. Similarly, IL-6 binds to its receptor and drives Th17 differentiation by activating RORγt and STAT3 (82, 83). Interestingly, TAK-242, a specific inhibitor of Toll-like receptor-4 (TLR4), was shown to alleviate UC by regulating macrophage polarization and Th homeostasis through the TLR4/JAK2/STAT3 signaling pathway (84).

## 4 Natural products involved in the regulation of JAK/STAT signaling in UC

### 4.1 Glycosides

**Figure 3:**
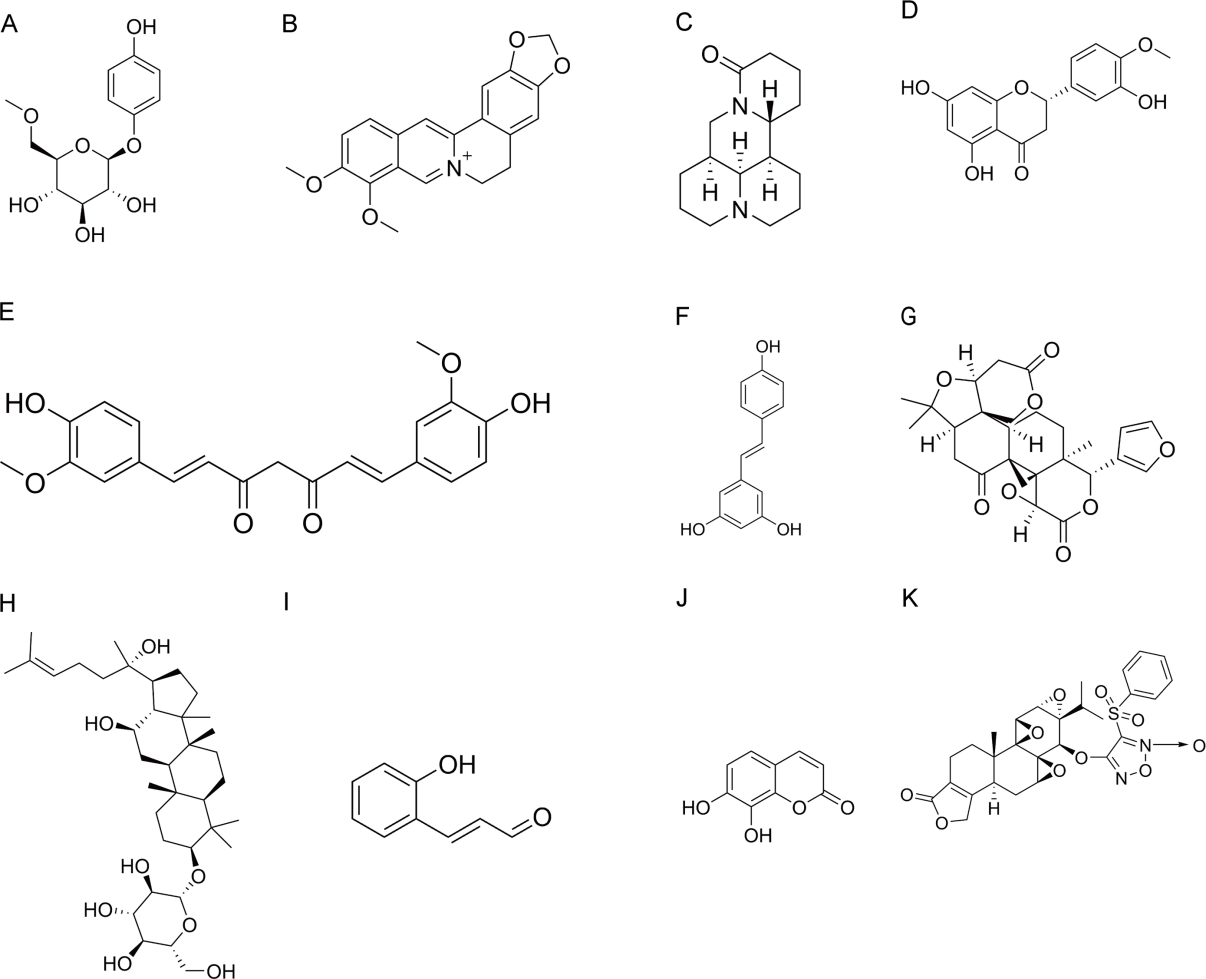
Chemical structures of natural products. **(A)** Arbutin. **(B)** Berberine. **(C)** Matrine. **(D)** Hesperetin. **(E)** Curcumin. **(F)** Resveratrol. **(G)** Limonin. **(H)** Ginsenoside Rh2. **(I)** 2′-Hydroxycinnamaldehyde. **(J)** Daphnetin. **(K)** ZT01.

Arbutin (molecular formula: C_12_H_16_O_7_, molecular weight: 272.25) is a glycoside compound mainly extracted from the leaves of arbutus. The chemical structure of arbutin is shown in **Figure 3**. Arbutin is a hydroquinone glucoside, with two different configurations: α and β arbutin. Compared to α-arbutin, β-arbutin is more frequently found in nature and typically occurs in higher concentrations in plants. β-arbutin has been widely researched for its whitening, anti-inflammatory, antimicrobial, antioxidant, and anticancer properties (85). Arbutin has been reported to significantly down-regulate the levels of inflammatory cytokines (IL-1β, IL-6, and TNF-α), iNOS, and cyclooxygenase-2 (COX-2) in colitis mice (86). In addition, arbutin remarkably inhibited the phosphorylation of JAK2 and STAT3 and suppressed IECs apoptosis, thereby improving barrier function (**Table 1**). *In vivo* experiment demonstrated that p-JAK2 expression was significantly inhibited by arbutin and AG490, a JAK2 inhibitor (86). No additional therapeutic efficacy was observed with the combination of arbutin and AG490. *In vitro* experiment showed that the inhibitory effect of arbutin on p-STAT3 and inflammatory factors (TNF-α and IL-6) was significantly reversed by AG490, further suggesting that arbutin may be a potential JAK2 inhibitor. These results indicated that the effect of arbutin on JAK was primary rather than secondary to broader anti-inflammatory effects. Interestingly, a recent study found that arbutin also inhibited the formation of neutrophil extracellular traps and increased the diversity and abundance of gut microbiota (87).

**Table 1:** Summary of natural compounds involved in the regulation of JAK/STAT signaling in UC.

Phytochemicals	Type	Experimental model	Mechanisms	References
Arbutin	Glycoside	DSS-induced UC mice, LPS-stimulated IEC-6 and RAW264.7 cells	↓TNF-α, IL-1β, and IL-6, ↓iNOS and COX2, ↑Bcl2, ↓MLCK, ↓p-JAK2, p-STAT3, and SOCS3	(86)
*Chrysanthemum* polysaccharide	Polysaccharide	TNBS-induced colitis rats	↓TNF-α, IFN-γ, IL-6, and IL-1β, ↓MDA and MPO, ↑SOD, ↓p-p65, TLR4, p-STAT3, and p-JAK2	(94)
Tetrastigma hemsleyanum polysaccharide	Polysaccharide	DSS-induced UC mice, Caco-2 cells induced by LPS in combination with IL-6	↑Claudin-1, ↓IL-6, TNF-α, MCP-1, and IFN-γ, ↓IL-17A, ↑Foxp3, ↓p-JAK2, JAK2, p-STAT3, and STAT3, ↑SOCS1	(97)
Aloe polysaccharide	Polysaccharide	TNBS-induced colitis rats, HT-29 cells stimulated by TNF-α and LPS	↓IL-6, ↓p-JAK2, JAK2, p-STAT3, and STAT3	(7)
Berberine	Alkaloid	DSS-induced UC mice	↓NLRP3, ASC, and cleaved caspase-1, ↓IL-1β, IL-6, and IL-18, ↑ZO-1, E-cadherin, occludin, claudin-1, and MUC2, ↓OSM and OSMR, ↓p-JAK1, p-JAK2, p-STAT1, and p-STAT3	(112)
Matrine	Alkaloid	DSS-induced NCM460 cells	↓TNF-α, IL-1β, IL-2, and IL-6, ↓MPO and NO, ↓Bax and cleaved caspase-3, ↑Bcl-2, ↓p-JAK2/JAK2 and p-STAT3/STAT3	(116)
Hesperetin	Flavonoid	TNBS-induced colitis rats	↑GSH and SOD, ↓NO content, ↓IL-6, NF-κB, RAGE, and TNF-α, ↓p-JAK2 and p-STAT3, ↑SOCS3	(117)
Curcumin	Polyphenol	DSS-induced UC mice	↓IL-7, IL-15, and IL-21, ↓JAK1, STAT5, and p-STAT5, ↑PIAS1	(122)
			↓p-STAT3, ↓DNA-binding activity of STAT3 dimers, ↓MPO, IL-1β, and TNF-α	(123)
		TNBS-induced colitis mice	↓p-JAK2, p-STAT3, and p-STAT6, ↑SOCS1, SOCS3, and PIAS3, ↓Activation of dendritic cells	(119)
Resveratrol	Polyphenol	DSS-induced UC mice, HCT116 cells	↓IL-6, IL-1β, and TNF-α, ↑ IL-10, ↓O-GlcNAcylation, ↓p-JAK2 and p-STAT3, ↓NOS2 and COX2	(133)
Ginsenoside Rh2	Terpenoid	DSS-induced UC mice, IL-6-stimulated NCM460 cells	↓TNF-α, IL-6 and IL-1β, ↓p-STAT3, ↓miR-214, ↑PTEN	(138)
Limonin	Terpenoid	DSS-induced UC mice, IL-6-stimulated NCM460 cells	↓IL-6 and TNF-α, ↑IL-10, p-STAT3, ↓miR-214, ↑PTEN and PDLIM2	(140)
2′-Hydroxycinnamaldehyde	Other types	DSS-induced UC mice, LPS-treated FHC cells	↓TNF-α, IL-6 and IL-1β, ↑IL-10, ↑ZO-1, occludin, Bcl-2, E-cadherin, and Claudin-3, ↓Bax, ↓p-STAT3 and the translocation of STAT3 from cytoplasm to nucleus	(145)
Daphnetin	Other types	DSS-induced mice, LPS-challenged Caco-2 cells	↓TNF-α, IFN-γ, IL-6, and IL-1β, ↑ZO-1, occludin, and BCL-2, ↓Bax and cleaved caspase 3, ↓MDA and SOD, ↓JAK2 and STAT3	(152)
ZT01	Other types	DSS-induced UC mice, BMDMs stimulated by LPS and IFN-γ	↑ZO-1 and E-candherin, ↓p-JAK1, p-JAK2, p-STAT1, and p-STAT3, ↓the differentiation of Th1 and Th17 cell, ↓the proinflammatory macrophage phenotype polarization	(154)

The symbol “↓” indicates inhibition, while “↑” represents promotion.

### 4.2 Polysaccharides

*Chrysanthemum morifolium* Ramat (Juhua), as a medicinal and edible homeopathic plant with strong heat-removing and detoxifying abilities, has long been widely used for the treatment of various diseases, including influenza, colitis, stomatitis, cardiovascular diseases, and various chronic diseases (88). Polysaccharide is one of the key active components in *Chrysanthemum morifolium* Ramat. It exhibits anti-inflammatory, antioxidant, immunomodulatory, anti-cancer, hepatoprotective, and gastrointestinal function regulatory effects (89, 90). The monosaccharides of *Chrysanthemum* polysaccharides (CP) mainly include galactose, glucose, arabinose, and mannose (89). Several studies have reported that the biological activity of CP is related to their chemical propertiesm, molecular weights, and monosaccharide composition (91, 92). The high antioxidant activity of snow CP is partly attributed to the low molecular weight and high content of unmethylated galacturonic acid (93). In rats with 2, 4, 6-trinitrobenzene sulfonic acid (TNBS)-induced colitis, CP reduced the expression of proinflammatory cytokines and blocked the phosphorylation of STAT3 and JAK2, resulting in significant relief of colitis (94). Furthermore, CP influenced biomarkers and metabolic pathways in plasma and urine. Although this study found that CP decreased the expression of IL-6/JAK2/STAT3 pathway-related proteins and mRNAs, it was not clear whether the effect of CP on JAK/STAT was primary or secondary to broader anti-inflammatory actions. For a deeper understanding of its mechanism of action, further experiments need to be designed to distinguish the primary and secondary effects. For example, after treatment with specific pathway inhibitors or activators, the changes in the effects of CP on the JAK/STAT pathway and inflammatory response should be observed.

*Tetrastigma hemsleyanum* Diels et Gilg (Sanyeqing) is a traditional herb native to China. Its whole plant is medicinally used and it is of great concern for its remarkable medicinal value. In particular, it shows strong potential in anti-inflammatory, antibacterial, and antiviral properties (95). *Tetrastigma hemsleyanum* Diels et Gilg is rich in active substances such as flavonoids, phenolic acids and polysaccharides. *Tetrastigma hemsleyanum* polysaccharide (THP) consists of various monosaccharides, mainly including galactose, glucuronic acid, mannose, glucose, rhamnose, and arabinose (96). THP has shown great potential in the treatment of UC (97, 98). THP reduces the expression of the proinflammatory cytokines IL-6, TNF-α, and IL-17 and promotes the regulatory factors forkhead box protein P3 and Tregs (97). Importantly, it exerts anti-inflammatory effects by promoting SOCS1 expression and inhibiting JAK2/STAT3 signaling (97). Additionally, THP elevates levels of tight junction proteins in colonic tissues and decreases colonic permeability, improving the intestinal mucosal barrier. Interestingly, it can also modulate the gut microbiota structure and corresponding short-chain fatty acid metabolites in mice with IBD (98). Notably, although most studies used p-JAK2 and p-STAT3 as efficacy biomarkers, they did not distinguish whether natural products inhibited them directly or regulated them indirectly through upstream cytokines or microbiota-derived metabolites. The specific mechanism by which natural products regulate JAK/STAT signaling is currently not precise enough. Follow-up studies could construct STAT knockdown models by CRISPR/Cas9 technology to verify the specific action targets of active ingredients.

Moreover, relevant upstream regulators can be knocked down or overexpressed to further explore the regulation mechanism of STAT3 phosphorylation by THP.

Aloe polysaccharide is an active macromolecule extracted from Aloe vera. It shows excellent protection against ulcers and significantly prevents ulcer formation (99). *In vivo* and *in vitro* experiments have demonstrated that aloe polysaccharide effectively ameliorated apoptosis in colon tissue by inhibiting the JAK2/STAT3 signaling pathway (7). Meanwhile, aloe polysaccharide contributed to the reduction of IL-6 levels and restoration of colon length in TNBS-induced UC rats. Similarly, this study only used methods such as immunohistochemistry, Western blot, and RT-PCR to detect the expression level of the JAK/STAT pathway, with insufficient depth and specificity of the mechanism of action.

### 4.3 Alkaloids

Berberine (molecular formula: C_20_H_18_NO_4_^+^, molecular weight: 336.4), a natural pentacyclic isoquinoline alkaloid, is the most representative and abundant constituent of the TCM *Coptis chinensis* Franch (Huanglian). Berberine contains two benzene rings, an isoquinoline ring, and functional groups such as methoxy groups, which endow it with unique biological activities. In addition to its anti-inflammatory and antioxidant activities, berberine exhibits a variety of pharmacological effects, including anti-apoptotic, antitumor, hepatoprotective, and cardiovascular protective effects (100–102). Interestingly, it is widely known for its anti-inflammatory effects in inflammatory gastrointestinal diseases (103). In a double-blind phase I trial, berberine was demonstrated to improve colonic mucosal histologic scores in Chinese patients with UC (104). Furthermore, the Xijing Hospital of Digestive Diseases is currently undertaking a phase IV clinical trial to determine the impact of berberine on the annual recurrence rate of UC in remission (NCT02962245, ClinicalTrials.gov). Interestingly, a recent *ex vivo* study explored the synergistic effects of berberine, *Hericium erinaceus*, and quercetin, providing a more effective therapeutic option for UC patients. Their combination reduced the expression of proinflammatory cytokines and promoted the expression of the anti-inflammatory cytokine IL-10 in IBD tissues (105). Berberine has been discovered to relieve experimental colitis by altering the inflammatory response of immunological and epithelial cells, improving intestinal barrier function, and modulating intestinal microbiota (106–109). Oncostatin M (OSM) belongs to the IL-6 cytokine family and is primarily produced by activated macrophages, neutrophils, dendritic cells, and T cells (110). Previous studies have confirmed that recombinant OSM induces the activation of the JAK-STAT pathway via a heterodimeric receptor consisting of OSMR and gp130 (111). Importantly, berberine has been found to alleviate intestinal fibrosis by inhibiting the OSM-mediated JAK-STAT pathway and interfering with the interaction between intestinal stromal cells and immune cells (112). Furthermore, berberine was shown to inhibit M1 macrophage polarization and induce M2 macrophage polarization, by activating the IL-4-STAT6 signaling pathway, thereby exerting a therapeutic effect on UC (113).

Radix Sophorae Flavescentis is the dried root of *Sophora flavescens* Aiton (Kushen), belonging to the Leguminosae family. It is a promising traditional herb with the effect of clearing heat and dampness and has long been used to treat UC. Alkaloids and flavonoids are the main components of *Sophora flavescens* Aiton. Matrine is isolated from the roots of *Sophora flavescens* Aiton, *Sophora tonkinensis*, and *Sophora alopecuroides* (Kudouzi). Matrine is a tetracyclic quinolizidine alkaloid with the chemical formula C_15_H_24_N_2_O and a molecular weight of 248.36. Matrine exhibits a wide range of pharmacological activities, including analgesic, anticancer, anti-inflammatory, antiviral, antifibrotic, and immunomodulatory effects (114). Because of its anti-inflammatory and immunomodulatory properties, matrine has great potential in the treatment of UC (115). Apart from this, matrine improved the composition and function of intestinal microbiota in mice with dextran sulfate sodium (DSS)-induced colitis. It decreased the proportions of *Firmicutes*, *Bacteroidetes*, and *Proteobacteria*, increasing the relative abundance of *Lactobacillus* and *Akkermansia *(115). A recent study confirmed that matrine inhibited proinflammatory factors, MPO activity, NO production, and apoptosis, thus effectively alleviating UC (116). Furthermore, matrine was found to suppress the phosphorylation levels of JAK2 and STAT3, but did not affect the phosphorylation of STAT5.

### 4.4 Flavonoids

Hesperetin (molecular formula: C_16_H_14_O_6_, molecular weight: 302.28) is a naturally occurring flavonoid compound in citrus fruits and is widely found in various traditional herbal medicines such as grapefruit peel, orange peel, and tangerine peel. In TNBS-induced UC rats, hesperetin significantly enhanced glutathione levels and superoxide dismutase activity to reduce colonic oxidative stress, while significantly reducing NO levels (117). Hesperetin also mitigated the inflammatory injury by significantly decreasing IL-6 as well as inhibiting the expression of NF-κB, receptor for advanced glycation end products, and TNF-α. In addition, hesperetin significantly inhibited the phosphorylation of JAK2 and STAT3 and promoted the expression aof SOCS3, thereby alleviating colitis. As mentioned above, the present study did not clarify whether natural products directly inhibit JAK2/STAT3 phosphorylation through small-molecule binding or indirectly modulate this pathway through upstream cytokines. Natural products may affect JAK2 and STAT3 phosphorylation through different mechanisms in different studies. If all do not distinguish between direct and indirect regulation and only use them as biomarkers of efficacy, it will cause incomparability between the results of studies.

### 4.5 Polyphenols

Curcumin (molecular formula: C_21_H_20_O_6_, molecular weight: 368.4) is an active polyphenol obtained from the dry rhizomes of herbs such as turmeric and tulip. It is also considered one of the potential drugs for the treatment of UC (118). Curcumin could alleviate UC by inhibiting dendritic cell-mediated expression of proinflammatory factors (119), modulating Th17/Treg homeostasis (120), and regulating M1/M2 macrophage polarization (121). Interestingly, the regulation of memory T cell homeostasis by curcumin is associated with the inhibition of JAK1/STAT5 signaling activity (122). In addition, curcumin not only suppressed STAT3 phosphorylation and STAT3 dimer binding to DNA, but also significantly inhibited the expression of proinflammatory cytokines, consequently ameliorating UC (123). Another study revealed that curcumin inhibited the phosphorylation of JAK2, STAT3, and STAT6 and upregulated the expression of downstream proteins (SOCS1, SOCS3, and PIAS3) in TNBS-induced UC rats (119). Moreover, curcumin inhibits dendritic cell activation and restores immune homeostasis by modulating the JAK/STAT/SOCS signaling pathway, effectively treating colitis (119). A randomized, double-blind, placebo-controlled trial demonstrated that the herbal combination of curcumin-QingDai significantly reduced the Disease Activity Index (DAI) score in patients with active UC and effectively induced their response and remission (CLINICALTRIALS: gov ID: NCT03720002).

Resveratrol (3,5,4’‐trihydroxy‐trans‐stilbene) is a polyphenolic stilbenoid isolated from Veratrum grandiflorum and abundantly found in grapes, mulberries, peanuts, rhubarb and several other plants. It is a well-known antioxidant (124). Due to its planar stilbene motif, resveratrol exhibits relatively high hydrophobicity. As a result, it demonstrates a comparatively strong affinity for hydrophobic pockets and binding sites within proteins. Furthermore, the polar hydroxyl (OH) groups serve as both hydrogen-bond donors and acceptors. These groups are capable of establishing numerous interactions with amino acid side chains and backbone amide groups (125). Clinical and preclinical studies have demonstrated that resveratrol exerts protective effects in numerous disease models, including digestive diseases, cardiovascular diseases, diabetes, tumors, and neurodegenerative diseases, which may be related to its multi-targeting properties (126–129). Notably, resveratrol has been demonstrated to restore the homogeneity and diversity of gut microbiota to some extent in colitis mice (130). Moreover, dietary resveratrol attenuated the inflammatory status and down-regulated the expression of proinflammatory cytokines such as IL-2, IFN-γ, IL-1β, IL-6, and TNF-α in colitis mouse model (131). Among the known resveratrol targets, JAK-STAT signaling has received widespread attention (132). It was shown that increased O-linked N-acetylglucosamine modification (O-GlcNAcylation) of STAT3 upregulated the expression of proinflammatory cytokines such as IL-6, IL-1β, and TNF-α, while downregulating the level of the anti-inflammatory cytokine IL-10 and aggravating colitis in mice (133). In addition, the levels of COX-2 and iNOS were elevated. Encouragingly, resveratrol inhibited the O-GlcNAcylation of STAT3, thereby inhibiting its phosphorylation as well as the activity of JAK2/STAT3 pathway, and consequently alleviating IBD (133). Moreover, resveratrol induced Tregs in mice with colitis, which was dependent on the downregulation of miR-31 (134). Meanwhile, it suppressed inflammatory T cells (Th1 and Th17). A randomized, double-blind, placebo-controlled study showed that supplementation with 500 mg resveratrol for 6 weeks improved the quality of life and reduced colonic inflammation in UC patients (135). Unfortunately, resveratrol’s low bioavailability and poor water solubility restrict its therapeutic use. The stability and oral bioavailability of resveratrol should be improved by future research using different delivery methods and changes (136).

### 4.6 Terpenoids

Ginseng, a traditional herbal medicine, is the dried root of *Panax ginseng* C. A. Meyer., a plant of the family Wujiaceae. As a valuable medicinal herb, it has been used in China for more than 2,000 years. Ginsenoside Rh2 (molecular formula: C_36_H_62_O_8_, molecular weight: 622.9) is one of the active ingredients extracted from ginseng root. It possesses various pharmacological activities and has great potential in the treatment of UC (137). *In vivo* and *in vitro* experiments revealed that ginsenoside Rh2 effectively inhibited STAT3 phosphorylation and miR-214 expression (138). Ginsenoside Rh2 was found to indirectly suppress STAT3 phosphorylation by inhibiting the upstream cytokine IL-6.

Limonin (molecular formula: C_26_H_30_O_8_, molecular weight: 470.5) is a triterpenoid derived from citrus and possesses favorable anti-inflammatory and antiapoptotic effects. Limonin reduced the generation of proinflammatory cytokines TNF-α, IL-1β, and IL-6 as well as the expression of inflammatory proteins COX-2 and iNOS in the colonic tissues of mice with DSS-induced colitis (139). Moreover, limonin was found to ameliorate DSS-induced chronic colitis in mice by inhibiting the endoplasmic reticulum-stressed PERK-ATF4-CHOP pathway and NF-κB signaling (139). In addition to this, limonin also improved the prognosis of UC by downregulating p-STAT3/miR-214 levels (140).

### 4.7 Other types

2’-Hydroxycinnamaldehyde (HCA) (molecular formula: C_9_H_8_O_2_, molecular weight: 148.16) is an active component isolated from the stem bark of *Cinnamomum cassia* (Rougui) (141). HCA was proved to have anticancer, anti-inflammatory, antioxidant, and immunomodulatory effects (141–143). Interesting, HCA was screened as a natural STAT3 inhibitor (141, 144). A recent study showed that HCA directly binds to STAT3 and inhibits its activation (145). The hydroxyl group of HCA may interact with the protein-binding site of STAT3 via hydrogen bonding to enhance binding specificity. Thanks to this property, it inhibits inflammatory cytokine expression, reduces apoptosis of IECs, and attenuates intestinal mucosal barrier damage, thus effectively alleviating UC (145).

Daphnetin (molecular formula: C_9_H_6_O_4_, molecular weight: 178.14), a coumarin derivative isolated from the Daphne plant, is a natural compound with multiple therapeutic potential (146–148). Daphnetin possesses oxygen-containing heterocycles with a characteristic benzo-α-pyrone framework (149). The catechol moiety served as the crucial pharmacophore for the antioxidant activity of daphnetin (149). Apart from its antioxidant activity, daphnetin also exhibits diverse therapeutic potentials, including anti-inflammatory, analgesic, antibacterial, neuroprotective, hepatoprotective, nephroprotective, and anticancer activities (150). A previous study has demonstrated that daphnetin ameliorates colitis by regulating microbiota composition and TH17/Treg balance (151). A recent study reported that Daphnetin attenuated intestinal inflammation, oxidative stress, and apoptosis in UC, which was associated with the inhibition of REG3A-dependent JAK2/STAT3 signaling (152).

Triptolide, a natural diterpenetriepoxide which is isolated from *Tripterygium wilfordii* Hook F (Leigongteng), has prominent anti-inflammatory and immunosuppressive properties. ZT01 is a newly obtained tretinoin derivative with strong anti-inflammatory effects and low toxicity (153). ZT01 may be an attractive candidate for future development as an anti-UC drug. Importantly, ZT01 significantly inhibits T cell differentiation into Th1 or Th17 cell subsets and prevents macrophage polarization to an inflammatory phenotype by modulating the JAK/STAT signaling pathway (154).

## 5 Conclusion and perspective

The increasing incidence of UC has placed a heavy burden on the global health system. The JAK/STAT signaling pathway mediates the pathogenesis of UC to some extent. There are limitations to some of the current studies on the JAK/STAT pathway and UC. The sample sizes of the studies detecting the activation level of the JAK/STAT pathway in colon biopsies from UC patients are small. Although it is possible to obtain information on a specific patient group to some extent, it is difficult to fully reflect the real situation of the entire UC patient population and is prone to bias. Moreover, single-center studies may be affected by factors such as geography and medical level, which makes the generalizability of the findings questionable. Future studies need to expand the sample size, use multicenter studies, and deeply investigate the reasons for the differences in JAK/STAT pathway activation. In terms of findings, higher levels of JAK and STAT expression were detected in the inflamed colonic mucosa of UC patients compared to the uninflamed mucosa. However, the current study did not further investigate the reasons for this difference, whether it is genetic differences in individuals, living environment or other factors. The lack of in-depth analysis would limit a comprehensive understanding of the pathogenesis of UC.

In recent years, natural products have received extensive attention from the medical community. They have the advantages of multiple pathways and multiple targets. It has been demonstrated that herbal active ingredients alleviate UC through various pathways, such as targeting the JAK/STAT pathway to reduce intestinal inflammation, improving the function of IECs, regulating Th17/Treg balance, and modulating macrophage status. This review systematically summarized the recent advances in natural products targeting the JAK/STAT pathway to treat UC, including polysaccharides, alkaloids, polyphenols, terpenoids, flavonoids, glycosides, and other types of compounds. Natural products are potential candidates to treat UC by targeting the JAK/STAT pathway.

Nevertheless, there are many challenges. Firstly, the current studies on the targeting of the JAK/STAT pathway by natural products for the treatment of UC mainly focus on the animal and cellular experimental level, which cannot be fully equated with the immunohistopathology of UC patients. At present, there is insufficient research on the pharmacokinetic properties of many natural products, such as their absorption, distribution, metabolism, and excretion in the body. These uncertainties affect the design of standardized dosages and the formulation of dosing regimens. Furthermore, natural products such as resveratrol have poor water solubility and low bioavailability, making it difficult to make suitable dosage forms for clinical use. There is an urgent need to improve the stability, solubility, and bioavailability of natural products to overcome the transformation challenges. Besides, when herbal active ingredients are combined with other drugs, they may affect pharmacokinetics and pharmacodynamics through multiple pathways, but their specific mechanisms and links are difficult to be clearly defined. These limitations pose a great challenge to mechanism research and efficacy assessment in the translation process of TCM. Preclinical and clinical studies are needed to validate the safety and efficacy of herbal active ingredients for the treatment of UC. Secondly, crosstalk exists between the JAK/STAT pathway and other signaling pathways, which means that targeting only one of JAK or STAT may not be sufficient for significant therapeutic effects. Exactly how natural products interfere with the JAK/STAT pathway and whether they interact with other signaling has not been fully elucidated. Therefore, subsequent scholars still need to conduct profound research on the mechanism of herbal active ingredients in the treatment of UC. Finally, the vast majority of studies have been limited to the effect of natural products on the JAK/STAT pathway and have not analyzed the in-depth laws between their chemical structures and pharmacological activities. The structural features of phytochemicals may influence their specificity for JAK/STAT through factors such as molecular size, shape, functional groups, charge distribution, and conformational flexibility. Although the intrinsic laws have not been fully revealed, structure-activity relationship studies and molecular docking will provide important clues for understanding these interactions.
